# Early application of pulsed electromagnetic field in the treatment of postoperative delayed union of long-bone fractures: a prospective randomized controlled study

**DOI:** 10.1186/1471-2474-14-35

**Published:** 2013-01-19

**Authors:** Hong-fei Shi, Jin Xiong, Yi-xin Chen, Jun-fei Wang, Xu-sheng Qiu, Yin-he Wang, Yong Qiu

**Affiliations:** 1 Department of Orthopaedics, Nanjing Drum Tower Hospital, The Affiliated Hospital of Nanjing University Medical School, No. 321 Zhongshan Road, Nanjing, China

**Keywords:** Electromagnetic field, Delayed union, Fracture healing, Long-bone fracture

## Abstract

**Background:**

Pulsed electromagnetic field (PEMF) is reported to be an effective adjunct for the management of nonunion long-bone fractures. Most studies implement PEMF treatment after 6 months or longer of delayed union or nonunion following fracture treatment. Despite these variations in treatment, the early application of PEMF following a diagnosis of a postoperative delayed union has not been specifically analyzed. In this study, the outcomes of postoperative delayed union of long-bone fractures treated with an early application of PEMF were evaluated as compared with a sham-treated control group.

**Methods:**

In this prospective, randomized controlled study, a total of 58 long-bone fracture patients, who presented with delayed union of between 16 weeks and 6 months, were randomly split into two groups and subjected to an early application of PEMF or sham treatment. Clinical and radiological assessments were performed to evaluate the healing status. Treatment efficacy was assessed at three month intervals.

**Results:**

Patients in the PEMF group showed a higher rate of union than those in the control group after the first three months of treatment, but this difference failed to achieve statistical significance. At the end of the study, PEMF treatment conducted for an average of 4.8 months led to a success rate of 77.4%. This was significantly higher than the control, which had an average duration of 4.4 months and a success rate of 48.1%. The total time from operation to the end of the study was a mean of 9.6 months for patients in the PEMF group.

**Conclusions:**

Fracture patients treated with an early application of PEMF achieved a significantly increased rate of union and an overall reduced suffering time compared with patients that receive PEMF after the 6 months or more of delayed union, as described by others.

## Background

Despite recent improvements in fracture management, delayed union and nonunion remain as intractable complications following surgical reduction and fixation of long-bone fractures. It is estimated that 5–10% of all fractures show impaired healing [1]. Surgical management is usually preferred in the treatment of an established nonunion, especially in those fractures that are accompanied by infection, deformity, shortening or bony defect. Otherwise, nonsurgical methods are considered for delayed union to facilitate osteogenesis, osteoinduction, as well as osteoconduction and thus stimulate the healing process [2,3]. Among the reported therapeutic methods, the use of biophysical interventions, such as pulsed electromagnetic field (PEMF) therapy, has attracted the attention of clinicians in the past decades, because of their noninvasive characteristics [4,5].

PEMF was introduced in the mid-1970s as a beneficial tool for fracture healing [6]. Although the mechanism remains poorly understood, PEMF provides an effective adjunct for the management of un-united long-bone fractures [7-10]. However, the indication and treatment strategies for the use of PEMF vary within the literature. The majority of investigators do not start PEMF treatment until an established nonunion is diagnosed [11-14], and others consider a late stage of delayed union (over 6 months after fracture) as the indication for its use [15-17]. Very few studies have addressed the early application of PEMF immediately after diagnosis of a delayed union (at about 16 weeks after fracture) [18], and no reports have specifically investigated the efficacy of the early application of PEMF.

Long-bone fracture healing has been recognized as an orchestration of prompt hematoma formation, inflammatory response, cell proliferation and differentiation, followed by a long-term process of ossification and remodeling [19]. Since the healing process is not considered to be accomplished in the case of a delayed union in orthopaedic terms, the early intervention of PEMF possesses the theoretical advantage of reactivating the biological process of bone repair, thereby facilitating fracture healing and possibly shortening the treatment duration. In the present study, the authors aimed to evaluate the efficacy of early-applied PEMF on postoperative delayed union of long-bone fractures. We hypothesized that the early application of PEMF in patients with delayed union might lead to an increased rate of fracture union compared with sham-treated patients. The outcomes of postoperative delayed union of long-bone fractures in patients treated with an early application of PEMF after the delayed union diagnosis were evaluated and compared with the placebo-treated controls.

## Methods

### Patients

**Figure 1:**
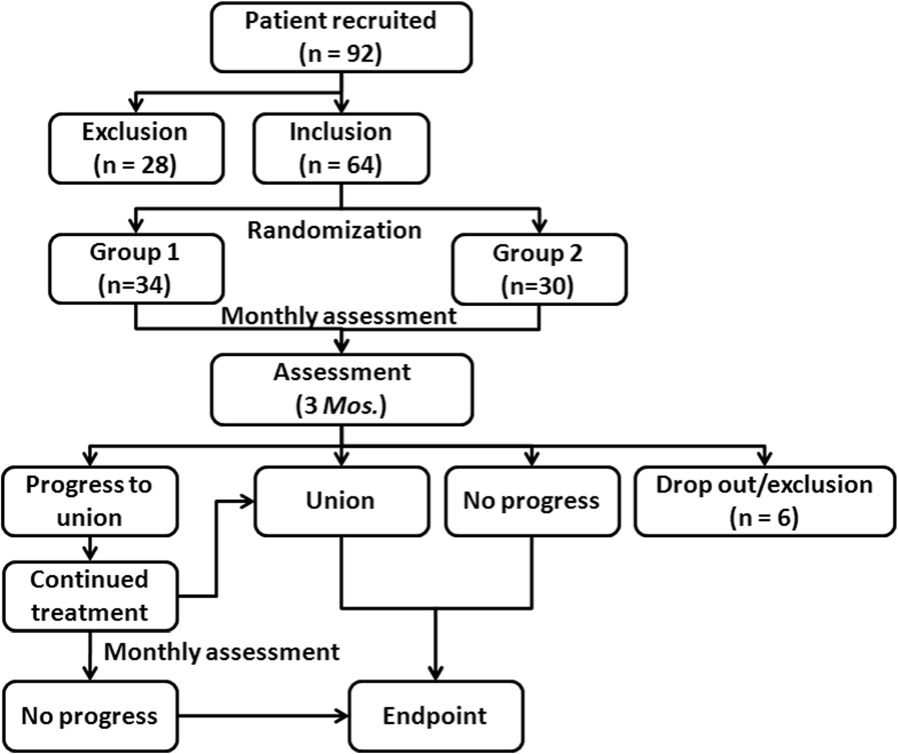
Flowchart of the study.

This prospective study was approved by the Medical Ethics Committee of Nanjing Drum Tower Hospital (Ref. No. 070321). A flowchart of the study is presented in Figure 1. Between April 2007 and September 2010, patients with postoperative delayed union of long-bone fracture were recruited from the outpatient clinic. During the baseline assessment, anteroposterior and lateral radiographs were taken to address the fracture healing status and the fixation method. Data on the demographic characteristics, comorbidity, medication history, lifestyle habits, fracture type, soft tissue condition were collected, as was information on the surgery and postoperative rehabilitation. Delayed union was defined as a failure to heal after at least 16 weeks and not more than 9 months following surgical reduction and fixation of the fracture [12,18]. Radiographically, healing failure was identified when callus bridging was not observed in more than three cortices on biplane radiographs. The exclusion criteria consisted of implant loosening or failure, infection, established nonunion (healing failure after more than 9 months, without any clinical or radiographic sign of progression to union within the last 3 months) [20], a fracture gap greater than 5 mm, and the presence of the implant within the fracture gap [11]. Patients with metabolic disorders were excluded as were those patients who received medications that could affect fracture healing [18,20].

The authors had intended to initiate intervention 16 weeks after fracture for each patient, but not all patients were referred to the clinic in time. Therefore, patients were included in the study if they were enrolled between 16 weeks and 6 months postoperatively. A power analysis was conducted to estimate the sample size, with reference to a previously reported randomized controlled trial that achieved a union rate of 89% in PEMF-treated tibial nonunion cases compared with a 50% union rate in the sham-treated controls [13]. To detect the similar change in union rate with 80% power in our study, we required more than 48 patients.

### Interventions

**Figure 2:**
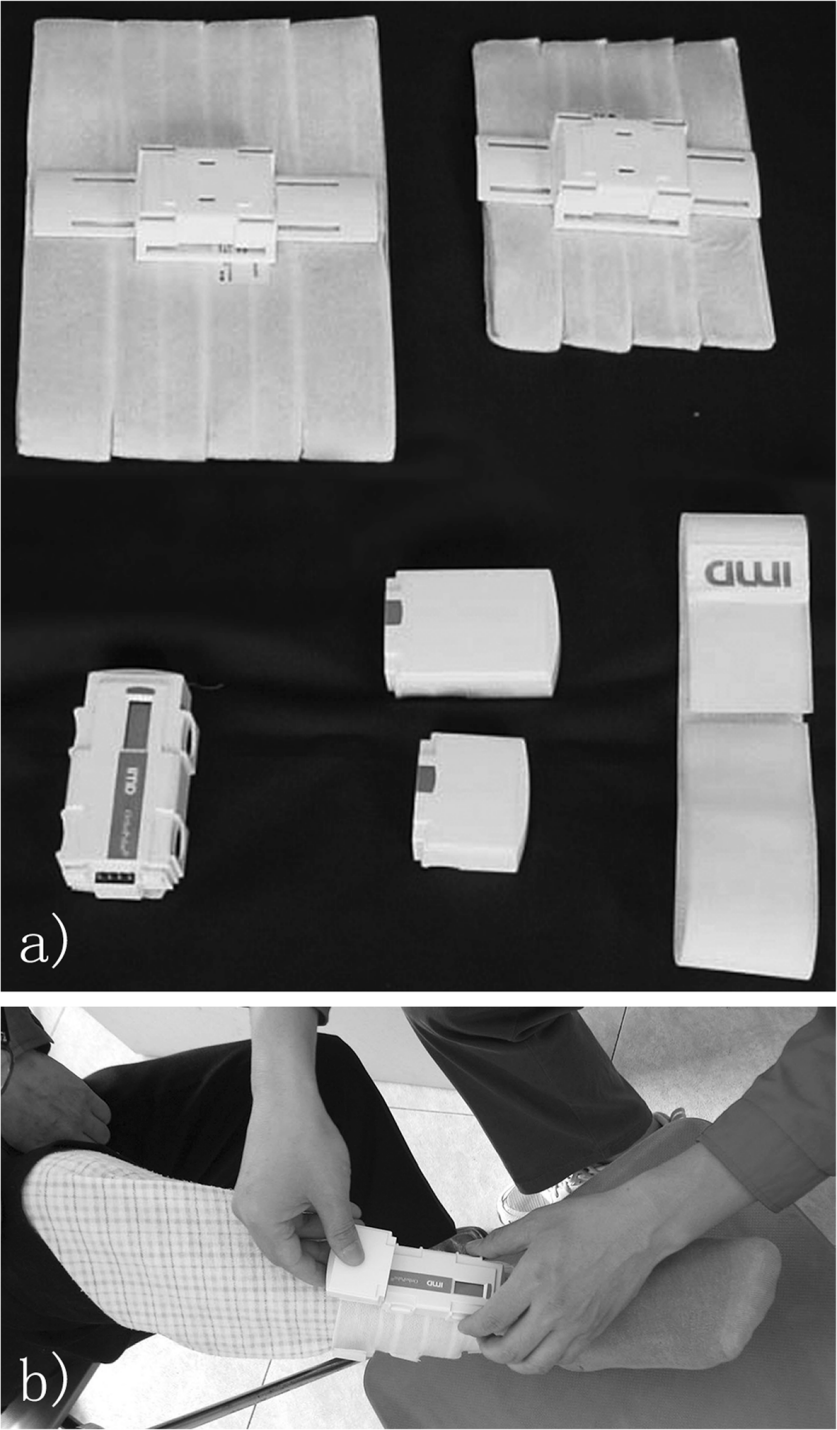
**The portable treatment equipments used in the study. **(**a**) A set of Orthopulse^® ^II stimulator consisted of different sizes of coils, signal generator, batteries, and removable fixation band; (**b**) Patient in Group 1 received pulsed electromagnetic field treatment with the coil centered over the fracture site.

Once included in the study, the patient was blindly assigned into the PEMF treatment group (Group 1) or the control group (Group 2) according to randomly generated numbers. In Group 1, PEMF treatment commenced immediately after enrollment. An electromagnetic field was delivered through a coil (Orthopulse^®^ II, OSSATEC, Uden, The Netherlands) centered over the fracture site for 8 h/day (Figure 2), with the signal specification adjusted according to Punt’s study [14]. In Group 2, the coil was applied for 8 h/day with a sham signal generator from the same manufacturer. Therefore, patients were blinded to the treatment. Protected weight bearing was encouraged unless it compromised the stability of the fractured area. All patients were requested to record their potential discomfort and the duration of the treatment. They were also asked to refrain from smoking, alcohol abuse, or additional forms of therapy during the study period. Biweekly contact through phone calls was performed by two research assistants to exclude patients with poor compliance.

### Outcomes

Clinical and radiological assessments were performed monthly following commencement of the treatment. Clinical evaluations of pain when stressed and motion at the fracture site were carried out by two senior surgeons (JFW and XSQ) independently, who were blinded to the grouping information. The consensus was derived from further discussion if necessary. Another two blinded surgeons (JX and YXC) reviewed the anteroposterior and lateral radiographs of the fracture to assess cortical bridging. Union was considered positive when there was no pain during joint stressing or during motion at the fracture site, and callus bridging was present for three out of four cortices on orthogonal radiographs [21]. Treatment was ceased in all patients when union was achieved or no radiographic progress to union was observed for a continuous three-month period (Figure 1).

### Statistical methods

Group demographics were compared using independent *t*-test or Fisher’s exact test. The successful rate of fracture union was calculated after three months of treatment and at the end of the study in each group, with the difference between groups compared with Fisher’s exact test. SPSS version 15.0 software (SPSS Inc, Chicago, IL) was used and the level of significance was set as 0.05.

## Results

During the study period, 92 patients with delayed union were recruited, with 64 patients meeting our inclusion criteria for early PEMF or sham treatment initiated 16 weeks and not more than 6 months postoperatively (Figure 1). Four patients dropped out after a short period of treatment, and another two patients, who received herbal supplements during the study, were excluded. The remaining 58 patients were included for statistical analysis. Patient demographics (Table 1) were comparable between the two groups, with no significant differences determined for patient age (*P* = 0.450), fracture site (*P* = 0.439), or method of fixation (*P* = 0.430). The original fracture sites included the humerus (5 cases), the ulna and/or radius (4 cases), the femur (24 cases), and the tibia (25 cases).

**Table 1:** Patient demographics and results

	**Treatment group**	**Control group**	**P Value**
No. of patients	31	27	
Age (*Yr.*)*	41.1 ± 14.5 (range 19 to 68)	38.4 ± 11.6 (range 20 to 62)	0.450
Fracture Site (No. of patients)			0.439
Femur	10	14	
Tibia	16	9	
Humerus	3	2	
Radius and/or Ulna	2	2	
Methods of Fixation			0.430
Plate	18	12	
Intramedullary Nail	13	15	
Elapsed Time before Treatment (*Mo.*)*	4.8 ± 0.9 (range 4 to 6)	5.1 ± 0.8 (range 4 to 6)	0.238
Duration of Treatment (*Mo.*)*	4.8 ± 2.3 (range 2 to 12)	4.4 ± 1.6 (range 2 to 7)	0.489
Rate of fracture union (3 *Mo.*)	38.7%	22.2%	0.256
Rate of fracture union (Endpoint)	77.4%	48.1%	0.029
Total Time from Operation to Endpoint (*Mo.*)*	9.6 ± 2.3 (range 7 to 17)	9.5 ± 1.5 (range 7 to 12)	0.849

* presented as mean ± SD.

**Figure 3:**
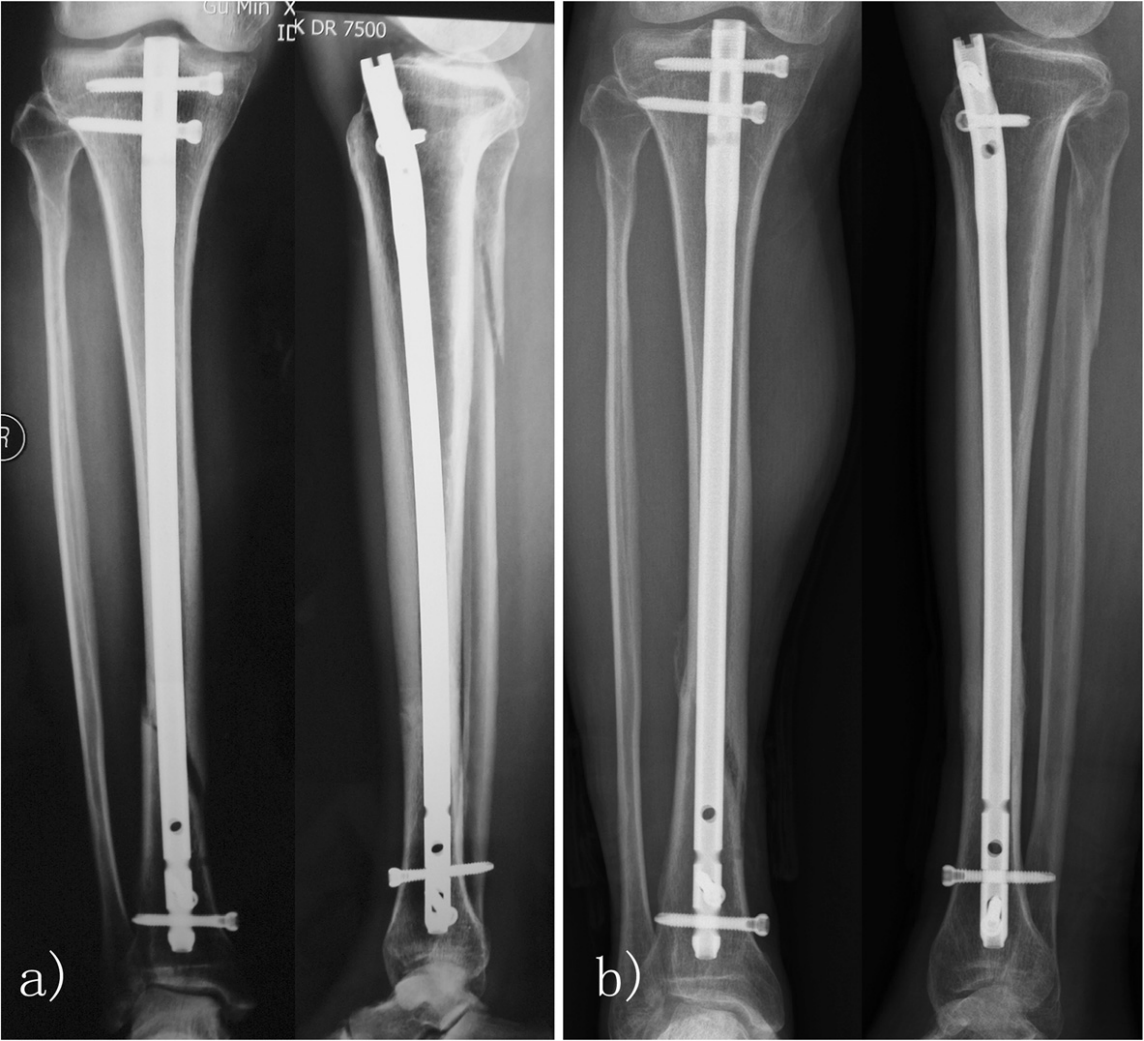
**Delayed union of tibia fracture treated with PEMF. **(**a**) A delayed union of tibia fracture was observed in a 65-year-old male patient following close reduction and intramedullary fixation 16 weeks ago. PEMF treatment was initiated; (**b**) Fracture union was observed after 3 months of treatment.

**Figure 4:**
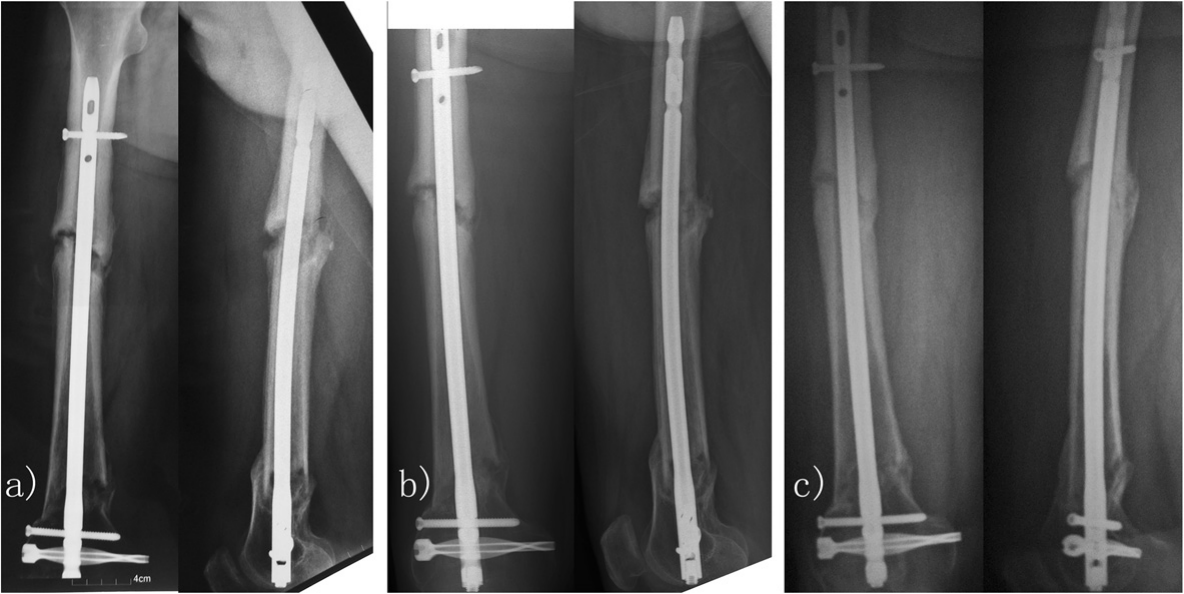
**Delayed union of femoral fracture treated with PEMF. **(**a**) PEMF treatment was started in a 59-year-old male patient who received reduction and intramedullary fixation 5 months ago; (**b**) Radiographies showed progress to union following 3 months of treatment; (**c**) Fracture united after 8 months of treatment.

A total of 31 patients received PEMF treatment, whilst the remaining 27 cases were assigned to the control group (Table 1). Before treatment, the average elapsed time since fracture operation were 4.8 months and 5.1 months in the two groups, respectively (*P* = 0.238). Following three months of treatment, 12 cases achieved union with a success rate of 38.7% (95% confidence interval (CI), 0.21 to 0.57) in Group 1 (Figure 3). Meanwhile, the fracture union success rate was 22.2% (6 out of 27, 95% CI, 0.08 to 0.42) for Group 2, which was slightly lower than that for Group 1 (*P* = 0.256), but not statistically significant. The relative risk of fracture union was 1.74 (95% CI, 0.76 to 4.01). Radiographic progress to union was observed in 17 patients in each of the groups, who subsequently received extended PEMF or sham treatment. At the end of the study, the average lengths of treatment were 4.8 months and 4.4 months in the two groups (*P* = 0.489), with a union rate of 77.4% (24 out of 31, 95% CI, 0.58 to 0.90) in Group 1 (Figure 4) compared with a union rate of 48.1% (13 out of 27, 95% CI, 0.28 to 0.68) in Group 2 (*P* = 0.029, Table 1). The relative risk of fracture union was 1.61 (95% CI, 1.04 to 2.48). The total times from operation to the end of the study were averaged at 9.6 months and 9.5 months in Group 1 and Group 2 respectively (*P* = 0.849). No discomfort was reported by the patients in either group during treatment.

## Discussion

In this randomized controlled study, we investigated, for the first time, the clinical efficacy of the early application of PEMF treatment in postoperative delayed union of long-bone fractures. Following three months of PEMF treatment, patients showed a higher rate of union (38.7%) than the sham-treated patients (22.2%), but this difference failed to achieve statistical significance. At the end of the study, PEMF treatment, conducted for an average duration of 4.8 months, led to a success rate of 77.4%, which is significantly higher than that in the control group (48.1%).

Clinically, the concepts and techniques surrounding the surgical management of long-bone fractures have evolved rapidly in recent decades. By comparison, the ensuing individual progress of fracture healing, in terms of biological and mechanical changes after surgery, has been poorly examined, despite the impaired healing rate of 5-10% in long-bone fracture patients. Among the multidisciplinary approaches explored to treat delayed union and nonunion fractures, the majority of studies employ the use of invasive procedures, such as surgical debridement, bone grafting and harvesting, or local injections [22,23], and hence, these procedures have been primarily examined in established nonunions. For delayed unions, noninvasive interventions, such as PEMF, are preferred before further invasive procedures are considered [4,24].

The original aim for this study was to instigate PEMF treatment immediately after the diagnosis of a postoperative delayed union (at 16 weeks after fracture). In our opinion, an earlier intervention is likely to be more effective because of the potentially deteriorated state of the biological environment after 16 weeks of delayed union or nonunion [25,26]. However in most published trials, PEMF stimulation was deferred until 6 months or later after fracture, with very few studies addressing the early application of PEMF in patients with delayed union. Sharrard conducted a randomized controlled trial with PEMF treatment initiated on patients with tibial delayed unions at 16 to 32 weeks after fracture [18]. Although the results revealed a significantly higher rate of union than the control, the authors did not specify the information and outcomes pertaining to the patients who received earlier intervention. A case series by Bassett addressed the effect of PEMF on 125 cases of delayed union and nonunion [27], with the earliest intervention started at four months after fracture. However, here again, the author only presented the overall success rate of the patients treated with PEMF within the nine month study period, without clarifying the impact of an early application of PEMF treatment. Similarly, in a report by Colson, there was a lack of consideration of the early effects of PEMF amongst 33 cases of long-bone delayed union or nonunion with treatment commenced from 2 to 120 months after fracture [28]. As such, our study provides pertinent evidence for the early application of PEMF on the delayed union of long-bone fractures.

The success rate following PEMF treatment in delayed union or nonunion varies dramatically (15.4–93.9%) across published studies due to different parametric settings and treatment strategies [28,29]. Considering studies with more than 30 subjects enrolled for PEMF treatment (a total of 12 studies, as summarized by Griffin), the average success rate was 80.1% (ranging from 67.6% to 93.9%) [10]. Using the same instrument as that used in our study, Punt examined a case series on established nonunions and achieved a success rate of 76–79% [14]. These results are comparable with the final success rate in our study (77.4%), demonstrating the similar stimulative effect of PEMF on delayed union, despite its earlier application in the present study. Therefore, our “sooner rather than later” hypothesis did not necessarily prevail for the clinical efficacy of PEMF. A recent report by Adie on the negative effect of PEMF on acute tibial shaft fractures further supports this [30].

Considering the treatment duration, no significant difference was observed between the groups in our study. However, the total time from fracture surgery to the end of PEMF treatment was obviously shortened in our study (9.6 months on average) compared with that in other studies who initiated PEMF stimulation after a postoperative window of 6 months, or longer in some cases (over 17.1 months in Heckman’s study, and 11.6 months in de Haas’s study) [15,16], not to mention the studies wherein PEMF treatment was applied in established nonunions. The early application of PEMF treatment, therefore, benefitted the patients by reducing the fracture suffering time. In clinical practice, PEMF treatment for delayed unions should be considered and initiated as early as possible, making patients fully aware of the success rate but also the increased cost.

At present, a definitive reason for the occurrence of a delayed union remains far from conclusive [31]. Both systemic and local factors are believed to be involved [23,32]. In our study, strict inclusion and exclusion criteria were set with reference to previously published clinical trials to rule out the interference of confounding variables such as metabolic disease, medication, smoking, alcohol abuse, infection, and unfavorable reduction or fixation from previous operations [11,18,20]. However, there were several factors constrained by practicality that may have influenced the outcome. For instance, the degree and extent of local damage caused by the accident or previous operation was difficult to trace. Further, patient activity levels, as a subject-related factor, could not be standardized during the study period, despite our recommendations for protected weight bearing. Another limitation of the present study was the relatively small numbers of patient for each fracture site or fixation method. We therefore could only draw an overall conclusion. Besides, serum biochemical markers were not measured in this study, which may potentially shed light on the biological mechanism of the early application of PEMF treatment.

## Conclusions

In conclusion, within the limitations discussed above, the early application of PEMF treatment promotes fracture healing and leads to a significantly increased rate of union compared with the sham treatment. Even though the final success rate in this study was not superior to that measured in other PEMF trials, we show that our patients benefitted from a reduced overall suffering time between fracture and repair.

## Competing interests

There’s no competing interests. No benefits in any form have been received or will be received related directly or indirectly to the subject of this article.

## Authors’ contributions

All authors read and agreed with the contents of the manuscript. JX and YXC participated in the study design and the radiographic outcome assessment. JFW and XSQ carried out the clinical outcome analysis. HFS was in charge of interpreting the data analysis and drafting the manuscript. YHW and YQ assisted in revising the manuscript. All authors read and approved the final manuscript.

## Pre-publication history

The pre-publication history for this paper can be accessed here:

http://www.biomedcentral.com/1471-2474/14/35/prepub
